# PECAM EMPs regulate apoptosis in pulmonary microvascular endothelial cells in COPD by activating the Akt signaling pathway

**DOI:** 10.18332/tid/146959

**Published:** 2022-05-03

**Authors:** Yuqin Zeng, Yiyang Zhao, Yan Chen, Shan Cai, Ping Chen

**Affiliations:** 1 Department of Pulmonary and Critical Care Medicine, The Second Xiangya Hospital, Central South University, Changsha, People’s Republic of China; 2 Research Unit of Respiratory Disease, Central South University, Changsha, People’s Republic of China; 3 Hunan Centre for Evidence-Based Medicine, Changsha, People’s Republic of China

**Keywords:** PECAM EMPs, chronic obstructive pulmonary disease, Akt signal pathway, apoptosis

## Abstract

**INTRODUCTION:**

Endothelial microparticles (EMPs) are partly associated with the progress of chronic obstructive pulmonary disease (COPD). We sought to measure the levels of EMPs in COPD patients and in human pulmonary microvascular endothelial cells (HPMECs) exposed to cigarette smoking extract (CSE) to elucidate the potential mechanisms of their action.

**METHODS:**

We obtained prospectively blood EMPs from 30 stable COPD patients and 20 non-COPD volunteers. EMP subpopulations were determined by flow cytometry in platelet-free plasma according to the expression of membrane specific antigens. Cell growth, proliferation, apoptosis and the expression of protein kinase B (Akt) in HPMECs after exposure to PECAM EMPs were assessed. After intervention with an antioxidant (Eukarion-134, EUK-134), apoptosis and the expression of Akt in HPMECs were also measured.

**RESULTS:**

Unlike those of MCAM EMPs, VE-cadherin, PECAM and E-selectin EMP values were significantly higher in the stable COPD patients than in the non-COPD volunteers (p<0.05). Only PECAM EMPs were higher in HPMECs exposed to CSE (p<0.05). Further, *in vitro* studies showed that the apoptosis rate and expression of cleaved caspase 3/9 in HPMECs increased in a dose- and time-independent manner with PECAM EMPs. The expression of phospho-Akt (p-Akt) decreased in a time-independent manner with PECAM EMPs (p<0.05). Compared with the control group, the early apoptosis rate of HPMECs was higher, and the expression of p-Akt was lower in both the PECAM EMP group and EUK-134 + PECAM EMP group (p<0.05). The apoptosis rate declined markedly, and the expression of p-Akt was higher in the EUK-134 + PECAM EMP group, compared with the PECAM EMPs group (p<0.05).

**CONCLUSIONS:**

The present results suggest that PECAM EMPs positively regulate apoptosis in HPMECs in COPD, likely by decreasing Akt phosphorylation and can be protected by antioxidants.

## INTRODUCTION

Chronic obstructive pulmonary disease (COPD) is defined as not fully reversible airflow obstruction caused by significant exposure to noxious particles or gases^1^. Cigarette smoking (CS) is the most common risk factor for COPD patients and plays a key role in endothelial cell injury in the development of barrier dysfunction, endothelial activation and inflammation, and apoptosis^2^. Increased endothelial cell apoptosis has been observed in CS extract (CSE)-exposed mice or COPD patients^3^. The potential mechanism underlying CS-induced endothelial apoptosis is rather complex^4^.

As we know, CS is closely associated with endothelial microparticles (EMPs) levels. EMPs (0.1–1.5 µm in diameter) are vesicles shed from endothelial plasma membranes into the circulation following cell activation and/or apoptosis, and contribute to inter-cellular communication^5^. EMPs are defined in terms of their membrane antigens, such as CD144 [vascular endothelial (VE)-cadherin], CD31 (platelet endothelial cell adhesion molecule, PECAM), CD146 (melanoma cell adhesion molecule, MCAM) and CD62E (E-selectin)^6^. Some of the released EMP subtypes have been observed to be significantly higher in patients with stable COPD than in non-COPD volunteers^7^. Compared with non-smokers, healthy smokers and COPD smokers have elevated levels of EMPs, which even remain high when COPD smokers quit smoking^8^. In smokers with normal spirometry but reduced diffusion capacity, EMPs with apoptotic characteristics (CD31) were increased^9^. Changes in the EMP subtype can demonstrate the physiological condition of the endothelial cells and the site of inflammation or apoptosis^10^. For example, VE-cadherin EMPs are elevated when endothelial activated, whereas PECAM EMPs are indicative of endothelial cell apoptosis^11^. However, the underlying mechanisms of EMPs involved in the apoptosis of endothelial cells induced by CS in COPD are still unknown.

The Akt (protein kinase B) signal pathway is implicated in cell apoptosis, inflammatory and promoting abilities of proliferation, and survival in COPD^12^. Akt belongs to the subgroup of serine-threonine protein kinases and can inhibit apoptosis by phosphorylation and inactivation of components of the apoptotic machinery^13,14^. A previous study showed that antioxidant interventions increase phosphorylation of Akt (p-Akt) in a model of diaphragm autophagy^15^. A salen-manganese mimetic of superoxide dismutase and catalase (Eukarion-134, EUK-134) is an antioxidant that can protect against muscle fiber atrophy through Akt pathway^16^. Moreover, EUK-134 attenuates asbestos-induced apoptosis in alveolar epithelial cells^17^. The Akt signal pathway is associated with the release of EMPs induced by CS, resulting in apoptosis and can be regulated by EUK-134.

Therefore, we hypothesized that EUK-134 can suppress apoptosis, which involves the release of EMPs in COPD through the Akt pathway. Firstly, we detected the numbers of EMPs released in COPD patients and non-COPD volunteers, and collected the number of EMPs in human pulmonary microvascular endothelial cells (HPMECs) exposed to CSE. Then, we investigated the role of EMPs and examined the function of Akt in apoptosis of HPMECs induced by CSE. Lastly, we measured the efficiency of EUK-134 in the Akt pathway in cell death.

## METHODS

### Participants

This study enrolled cases of COPD and control volunteers from the Second Xiangya Hospital of Central South University in China. The study was approved by an institutional review board from the Second Xiangya Hospital of Central South University. Written informed consent was obtained from all participants.

Thirty stable COPD patients (defined as a postbronchodilator FEV_1_/FVC <0.70 and with no exacerbation within the previous 3 months prior to sample collection1) and 20 healthy non-smoking volunteers without COPD, were enrolled in this study. All participants were aged ≥40 years without conditions known to be associated with an increase in circulating EMPs, including cardiovascular diseases such as hypertension, chronic renal failure, metabolic diseases such as diabetes mellitus, hyperlipidemia, and vasculitis^7,18^. Other lung diseases, pulmonary hypertension, prior lung resection, heart or lung transplant, cancer, allergy to gadolinium, claustrophobia and pregnancy were excluded.

### Pulmonary function test

We performed all spirometric maneuvers with the participants in a seated position, wearing a nose clip, and using a disposable mouthpiece (Hypair Medisoft, Belgium). The report quality was controlled based on the American Thoracic Society and European Respiratory Society criteria^19^.

### Collection of EMPs

*Blood samples*

All subjects fasted from 10 p.m. the previous day until the next morning. Venous blood of the antecubital fossa was extracted on an empty stomach and in the resting state. Preparation of EMP samples were carried out as previously described^9,10^. Blood was collected in 15 mL sodium citrate tubes and transferred to centrifuge tubes within 1 h, then centrifuged for 10 min (at 170g, 23°C) to prepare platelet-rich plasma and further centrifuged for 20 min (at 1500g, 23°C) to obtain platelet-poor plasma. We incubated 50 μL aliquots of platelet-poor plasma (45 min, 4°C) with 4 μL von Willebrand factor (vWF, #210905, R&D System, USA) to identify EMP from pulmonary capillary endothelium cells. Then 4 μL of fluorescein-conjugated anti-human PECAM (CD31+ FITC, #560984, BD Biosciences, USA), anti-human VE-cadherin (CD144+ FITC, #560874, BD Biosciences, USA), anti-human E-selectin (CD62E+ PE, #551145, BD Biosciences, USA) and anti-human MCAM (CD146+ PE, #561013, BD Biosciences, USA) were added separately to different tubes and incubated on ice for 30 min.

*Cell samples*

After treatment with 10% CSE, HPMECs supernatants were collected in 1.5 mL tubes by centrifugation for 5 min (at 1500g, 23°C) followed by centrifugation for 1 h (at 20000g, 4°C). Then, cells were resuspended in bovine serum albumin (BSA, Sigma, USA) buffer, and EMPs relative antibodies were added, as above, using correlating isotype control antibodies for correction. PECAM (CD31^+^) and CD31^-^ EMPs were sorted by flow cytometry and centrifuged 1 h (at 20000g, 4^o^C). Then, they were resuspended in EBM-2 (Lonza, USA) for the next study. PECAM EMPs were diluted to different concentrations (0.7×10^5^/mL, 1.4×10^5^/mL, 2.8×10^5^/mL, 5.6×10^5^/mL).

### Characterization of EMPs

The method of characterization of EMPs was conducted as previously described^7^. EMP subpopulations were determined by flow cytometry in platelet-free plasma according to the expression of membrane specific antigens: VE-cadherin EMPs: CD144+ (FITC) MPs; PECAM EMPs: CD31+ (FITC) MPs; MCAM EMPs: CD146+ (PE) MPs; and E-selectin EMPs: CD62E+ (PE) MPs. We defined EMPs derived from pulmonary capillary endothelial cells as von Willebrand factor (vWF)-negative EMPs because arterioles and venules in the lungs and endothelial cells in other organs are positive while alveolar capillaries are negative for vWF^20,21^. Briefly, pulmonary EMPs were defined as VE-cadherin (CD144+/vWF-) EMPs, PECAM (CD31+/vWF-) EMPs, E-selectin (CD62E+/vWF-) EMPs, and MCAM (CD146+/vWF-) EMPs.

EMPs were quantified by flow cytometry using Cell Quest-Pro software (FACS Arial III, BD Biosciences, USA) by investigators blinded to subject status. The detailed protocol is given in the Supplementary file.

### Primary cells and cell culture

HPMECs were purchased from ScienCells Research Laboratory (#3000, Carlsbad, CA) and were cultured in EGM-2 MV (Lonza, USA) supplemented with 5% FBS, 1% endothelial cell growth factor, and penicillin (100 IU/mL)/streptomycin (50 μg/mL). The cells were then placed in a humidified incubator, which had a constant temperature of 37°C and 5% CO_2_. The culture medium was changed 12 h after plating and again after 48–72 h.

### Preparation of CSE

CSE was prepared as previously described^22^. Briefly, one non-filtered Fu-Rong cigarette (per cigarette: tar 12 mg, nicotine 1.0 mg, carbon monoxide 14 mg, China Tobacco Hunan Industrial Co., Ltd., Changsha, China) was burned and the smoke passed through 25 mL of phosphate-buffered saline (PBS) via a vacuum pump. This product was supposed to be a 100% CSE solution, which would be adjusted to different concentrations. The aqueous smoke extract was filtered through a 0.22 μm membrane filter to remove particles and bacteria, and the pH of CSE was adjusted to 7.2–7.4 before use.

### Assessment of cell growth and proliferation in HPMECs

A3-(4,5-dimethylthiazol-2-yl)-2,5-diphenyltetrazolium bromide (MTT) colorimetric assay (Invitrogen, USA) was used to assess cell growth. Briefly, cells were cultured in a 96-well plate. A 20 μL volume of MTT medium was added to the cells for 4 h at room temperature, and then 200 μL DMSO (Sigma, USA) was added. The optical density was set at 545 nm (Bio-Rad, USA). Cells grown with no PECAM EMPs served as a control. The percentage growth rate was calculated.

Cell proliferation was evaluated using immunofluorescence staining as described previously^23^. Cells were cultured in 24-well plates and treated with 2.8×10^5^/mL PECAM EMPs at 0, 3, 6, 12, and 24 h. After treatment, the cell culture slides were incubated with the Ki67 monoclonal antibody (TA500265, OriGene, USA) at 4°C overnight. After washing, cells were incubated with cyanine 5-conjugated goat anti-mouse IgG (Thermo, USA) for 2 h at room temperature and then counterstained with 4', 6-diamidino-2-phenylindole (DAPI) for 5 min from light and detected by a fluorescence microscope (Axioshop40, Carl Zeiss, Germany). All experiments were repeated three times.

### Assessment of apoptosis in HPMECs

An AnnexinV-FITC apoptosis kit (BD FACSCalibur, San Jose, CA, USA) was used to assess apoptosis according to the manufacturer’s instructions. Briefly, after treated with CSE, PECAM EMPs, 20 μM EUK-134 (Abcam, Britain) or PECAM EMPs + 20 μM EUK-134, cells were harvested by centrifugation, washed twice with ice-cold PBS and binding buffer, and incubated with AnnexinV-FITC and propidium iodide (PI) in the dark for 30 min at room temperature. Then, apoptosis was detected by flow cytometry using a FACS Arial III (BD Biosciences, USA). Cells that stained negative for PI and positive for annexin V were considered early apoptotic. Cells staining positive for PI and annexin V were considered late apoptotic or necrotic cells. Each experiment was performed in triplicate and repeated independently at least three times.

### Caspase activity assays

The activation of caspase 3/8/9 was evaluated by the caspase 3/8/9 activity assay kit (Beyotime Institute of Biotechnology, China) according to the standard protocol. To measure the activity of caspase 3/8/9, 50 μg of total proteins per well were incubated separately with Ac-DEVD-pNA, Ac-IETD-pNA, and Ac-LEHD-pNA substrates to develop a yellowish color. The absorbance of the samples was measured at 405 nm with a spectrophotometric microplate reader (Bio-Rad, USA). The detailed protocol is given in the Supplementary file.

### Western blotting analysis

Total proteins from HPMECs were extracted on ice using RIPA buffer (Applygen Company, Beijing, China) supplemented with protease inhibitors (Applygen Company, Beijing, China). The concentration of total proteins was measured using the BCA protein assay kit (Beyotime, Shanghai, China). Then, specified amounts of protein samples were separated by SDS-PAGE (Sigma, USA) and transferred onto PVDF membranes (Millipore, USA). The membranes were blocked with 5% non-fat dry milk in PBS containing 0.05% Tween (PBST) for 1h. Following blocking, these membranes were washed and incubated overnight at 4°C, with primary rabbit antibodies: anti-caspase-3 (1:1000, #14220, Cell Signaling Technology, USA), anti-caspase-9 (1:1000, #20750, Cell Signaling Technology, USA), anti-cleaved caspase-3 (1:1000, #9664, Cell Signaling Technology, USA), anti-cleaved caspase-9 (1:1000, #20750, Cell Signaling Technology, USA), anti-Akt (1:1000, #9272, Cell Signaling Technology, USA), anti-phospho-Akt (1:1000, #4058, Cell Signaling Technology, USA), and anti-β-actin (1:1500, #4970, Cell Signaling Technology, USA). Next, the membrane was washed three times and incubated with horseradish peroxidase-labeled goat-anti-rabbit IgG secondary antibody (1:3000, #7074, Cell Signaling Technology, USA) for 1 h at room temperature. After washing, the antigen-antibody complexes were visualized using enhanced chemiluminescence detection reagents (ECL, Thermo, USA) and the protein levels were quantified using Image J (National Institutes of Health, Bethesda, MD); β-actin was used to confirm equal protein loading and transfer.

### Real-time polymerase chain reaction (PCR)

The total RNA was extracted using TRIzol Reagent (Life Technologies) according to the manufacturer’s protocol. The RNA was reverse transcribed using the PrimeScript™ RT reagent Kit (TaKaRa, Dalian, China) and assayed using SYBRR® Premix Ex TaqII following the manufacturer’s instructions. Real-time PCR was carried out with the Step-one ABI Real-time RT-PCR system. The primer sequences were synthesized by Sangon Shanghai, China and were as follows: Akt, 5'-GGACAACCGCCATCCAGACT-3' (F) and 5'-GCCAGGGACACCTCCATCTC-3' (R); β-actin, 5'-GCACCACACCTTCTACAATGAG-3' (F) and 5'-GATAGCACAGCCTGGATAGC A- 3' (R).

### Statistical analysis

Descriptive statistics included the absolute and relative frequencies for categorical variables and the mean ± SD for numerical variables. Continuous variables were compared statistically using t-tests, and categorical variables were compared using the Manne-Whitney U test. ANOVA was used to compare multiple groups. We considered a p-value less than 5% to indicate statistical significance. All analyses were performed using PASW version 18.0 software (PASW, Inc., Chicago, Illinois). Because of the abnormal distribution of EMPs, Log conversion was performed before the univariable analysis. Multivariable linear regression analysis was performed to evaluate the impact of patient characteristics including age, smoking, and lung function on EMP numbers. Even though age and smoking were not relevant to EMP numbers in our study, we also added the two variables in the analysis, according to previous research^7^.

## RESULTS

### Characteristics of the study population

In total, 30 COPD patients and 20 healthy non-smoking volunteers without COPD were enrolled in our study. All demographic parameters are shown in Table 1. COPD patients had lower lung function and higher C-reactive protein compared with non-COPD volunteers (p<0.05). There was no difference between COPD patients and non-COPD volunteers in age, sex, BMI, and white blood cell count (p>0.05).

**Table 1:** The baseline clinical characteristics of COPD patients and non-COPD volunteers

*Characteristics*	*Non-COPD (N=20) Mean ± SD*	*Stable COPD (N=30) Mean ± SD*	*p*
Age (year)	68.9 ± 5.6	69.2 ± 6.7	0.869
Male, n (%)	14 (70)	25 (83)	0.221
BMI (kg/m2)	28.63 ± 5.21	26.89 ± 5.55	0.271
Smoking (pack-years)	0	32.91 ± 10.45	-
**Lung function**			
FEV_1_, % predicted	91.42 ± 7.11	68.50 ± 8.69	<0.001*
FEV_1_/FVC (%)	80.51 ± 8.63	64.31 ± 11.93	<0.001*
WBC (×109/L)	6.23 ± 1.82	6.99 ± 2.61	0.264
CRP (mg/L)	3.01 ± 1.24	4.35 ± 3.12	0.041*

BMI: body mass index. FEV_1_: forced expiratory volume in 1 second. FVC: forced vital capacity. WBC: white blood cell. CRP: C-reactive protein. T-test was used to compare the difference between groups.

* Compared with the non-COPD volunteers, p<0.01.

### EMP numbers and continuous variables in the study population

When compared with the non-COPD volunteers, the PECAM, VE-cadherin and E-selectin EMP numbers were significantly higher in the stable COPD patients (p<0.05). However, there was no difference in MCAM EMP numbers (p=0.254), in agreement with Takahashi et al.^7^. According to the results of univariable and multiple linear regression analysis, PECAM, VE-cadherin, and E-selectin EMPs were significantly correlated with lung function parameters (p<0.001). Only PECAM EMPs were associated with smoking (|r|=0.210, p=0.002) (Tables 2 and 3).

**Table 2:** Relationships between EMP numbers and various variables in COPD patients and non-COPD volunteers (univariable analysis)

*Variables*	*Log (VE-cadherin EMPs) ^a^*		*Log (PECAM EMPs)*		*Log (E-selectin EMPs)*	
	*r*_s_	*p*	*r*_s_	*p*	*r*_s_	*p*
Age (years)	0.174	0.067	0.160	0.080	0.163	0.076
BMI (kg/m2)	-0.128	0.097	-0.101	0.169	-0.122	0.101
Smoking (pack-years)	0.149	0.082	0.210	0.002*	0.197	0.069
**Lung function**						
FEV_1_/Pre (%)	-0.613	<0.001*	-0.479	<0.001*	-0.523	<0.001*
FEV_1_/FVC (%)	-0.470	<0.001*	-0.577	<0.001*	-0.507	<0.001*
WBC (×109/L)	0.107	0.168	0.027	0.309	0.128	0.159
CRP (mg/L)	0.024	0.317	0.025	0.313	0.019	0.322

BMI: body mass index. FEV_1_: forced expiratory volume in 1 second. FVC: forced vital capacity. WBC: white blood cell. CRP: C-reactive protein. Univariate logistic regression analysis was used,

* p<0.05.

a Because of abnormal distribution in EMPs, log-transformation was used.

**Table 3:** The multiple logistic regression analysis of EMPs

*Variable*	*Log (VE-cadherin EMPs)* ^a^			*Log (PECAM EMPs)*			*Log (E-selectin EMPs)*		
	*β*	*95% CI*	*p*	*β*	*95% CI*	*p*	*β*	*95% CI*	*p*
Age (years)	0.010	(-0.077 – 0.079)	0.836	0.034	(-0.065 – 0.134)	0.566	0.011	(-0.088 – 0.109)	0.692
Smoking (pack-years)	0.049	(-0.026 – 0.124)	0.263	-0.065	(-0.029 – -0.110)	0.017*	0.044	(-0.045 – 0.127)	0.303
FEV_1_/Pre (%)	-0.147	(-0.233 – -0.060)	<0.001*	-0.135	(-0.196 – -0.072)	<0.001*	-0.263	(-0.332 – -0.193)	<0.001*
FEV_1_/FVC (%)	-0.067	(-0.086 – -0.048)	<0.001*	-0.123	(-0.185 – -0.061)	<0.001*	-0.231	(-0.301 – -0.161)	<0.001*

FEV_1_: forced expiratory volume in 1 second. FVC: forced vital capacity. WBC: white blood cell. CRP: C-reactive protein. Multiple logistic regression analysis was used,

* p<0.05.

a Because of abnormal distribution in EMPs, log-transformation was used.

### PECAM EMPs released by HPMECs increased after exposure to CSE

**Figure 1:**
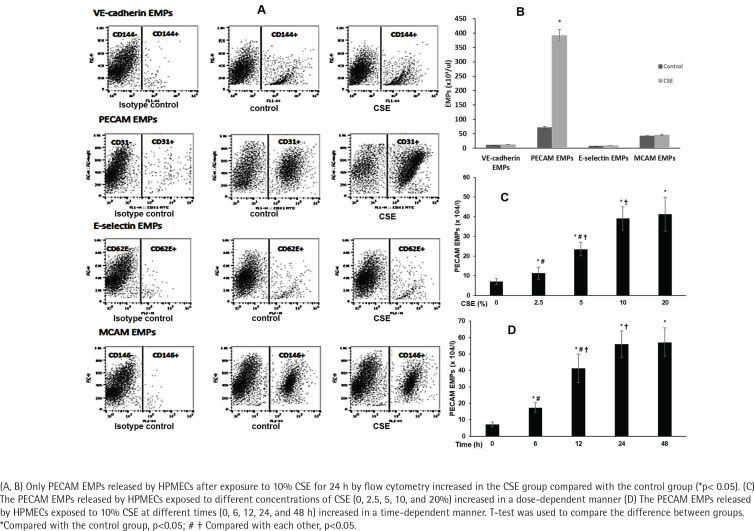
The effect of EMPs numbers released by HPMECs after exposure to CSE

To further confirm the release of EMPs induced by smoking *in vitro*, we enumerated the EMPs released by HPMECs exposed to 10% CSE after 24 h by flow cytometry (Figure 1). Only PECAM EMPs produced by HPMECs were increased in the CSE group compared with the control group (Figures 1 A and B). In addition, when exposed to higher concentrations of CSE (≥2.5%) for 12 h, the PECAM EMPs were released by HPMECs in a dose-dependent manner (Figure 1 C). Then, after treatment with 10% CSE at different times (0, 6, 12, 24, and 48 h), the numbers of PECAM EMPs released by HPMECs increased gradually up to 24 h in a time-dependent manner. There was no significant difference in PECAM EMP numbers between 24 h and 48 h (Figure 1 D).

### Cell growth, apoptosis, and proliferation of HPMEC exposed to PECAM EMPs

To address whether PECAM EMPs would affect the function of HPMEC, we observed the cell growth and apoptosis of HPMEC exposed to different concentrations of PECAM (CD31^+^) EMPs for 24 h in comparison with non-PECAM EMPs (CD31^-^) and control group (the supernatant from the last centrifugation during EMPs extraction) (Supplementary file Figure S1 A–C). The cell growth rate of HPMECs decreased gradually and the apoptosis rate of HPMECs increased gradually compared with the control group when the cells were exposed to a higher concentration than 1.4×10^5^/mL PECAM EMPs (p<0.05, p<0.05). There was no significant difference in the cell growth rate and apoptosis rate of HPMECs in CD31^-^EMPs and 0.7×10^5^/mL PECAM EMPs compared with the control group (Supplementary file Figure S1 A–C; p>0.05, p>0.05).

To investigate the proliferation using Ki67 and DAPI staining, we co-cultured 2.8×10^5^/mL PECAM EMPs with HPMECs for different times (0, 3, 6, 12, and 24 h). After treatment, the Ki67-positive HPMECs were reduced gradually as the exposure time increased (Supplementary file Figure S1 D–E; p<0.05).

### Activity and expression of caspase 3/9 in HPMECs exposed to PECAM EMPs

Apoptosis is a mitochondrion-centered cell death which results in apoptosome formation, activation of caspase-8/9 and subsequent activation of effector caspases, such as caspase 3^24^. Therefore, we postulated that PECAM EMPs induced the apoptosis of HPMECs by activating the caspase family. To test this hypothesis, we measured the activity of caspase 3/8/9 by spectrophotometry (Supplementary file Figure S2 A). We observed that the activity of caspase 3 increased gradually and significantly in HPMECs after 3, 6, 12, and 24 h of exposure to PECAM EMPs compared with the control group (0 h), which was similar to the behavior of caspase 9 (p<0.05). By contrast, the level of caspase 9 reached its peak value at 12 h. However, there was no change in caspase 8 as the exposure time increased (p>0.05), suggesting that only caspase 3/9 may regulate apoptosis induction by PECAM EMPs.

To further examine the changes, we used western blotting to investigate the expression of caspase 3/9 in HPMECs exposed to 5.6×10^5^/mL PECAM EMPs at different times (Supplementary Figure S2 B–C). After treatment with PECAM EMPs 6 h later, the expression of cleaved caspase 3 increased gradually, while caspase 3 decreased slowly (p<0.05 vs control). Exposure to PECAM EMPs for 3 h increased the expression of cleaved caspase 9 (p<0.05 vs control), while caspase 9 decreased slowly after 6 h (p<0.05 vs control). Collectively, these results indicate that caspase 3/9 can regulate apoptosis in HPMECs exposed to PECAM EMPs.

### Expression of Akt in HPMECs exposed to PECAM EMPs

Several molecular and signaling pathways play a crucial role in regulating apoptosis, such as the caspase family and the Akt pathway. To demonstrate whether PECAM EMPs induced apoptosis through the Akt signaling pathway, we used western blotting to observe the expression of phospho-Akt (p-Akt) and total Akt (t-Akt) in HPMECs exposed to 5.6×10^5^/mL PECAM EMPs at different times (0, 3, 6, 12, and 24 h) (Supplementary file Figure S3 A). Compared with the control group (0 h), the expression of p-Akt decreased in the exposure group as the treatment time increased (p<0.05), whereas, the protein and mRNA expression of total Akt displayed no meaningful changes in the exposure group compared with the control group (Supplementary file Figure S3 B; p>0.05).

### Apoptosis and expression of Akt in HPMECs exposed to different interventions

Next, to evaluate the effect of antioxidants on Akt signal pathway regulation of apoptosis, we treated HPMECs with EUK-134 and then examined the changes in Akt and apoptosis (Supplementary file Figure S4). Compared with the control group, the early apoptosis rate of HPMECs was obviously higher and the expression of p-Akt was lower in the PECAM EMPs group and EUK-134 + PECAM EMPs group (p<0.05, p<0.05). The apoptosis rate declined markedly and the expression of p-Akt was higher in the EUK-134 + PECAM EMPs group compared with that in the PECAM EMPs group (p<0.05, p<0.05). There was no difference between the control and EUK-134 group (Supplementary file Figure S4 A–C; p>0.05), whereas there were no significant differences in the protein and mRNA expression of t-Akt between groups (p>0.05).

## DISCUSSION

**Figure 2:**
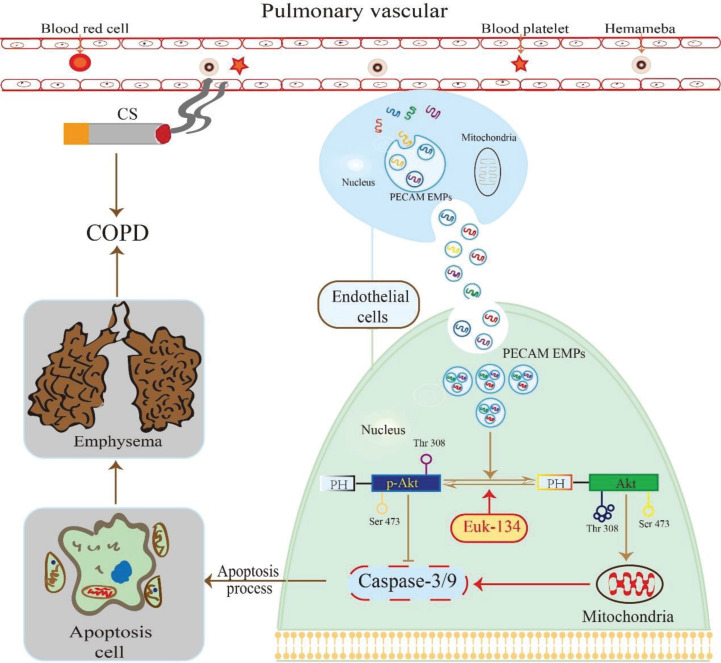
The mechanisms

We found that VE-cadherin EMPs, PECAM EMPs, and E-selection EMPs were more abundant in stable COPD patients and negatively correlated with lung function, while MCAM EMP numbers were not elevated. Only PECAM EMP numbers were higher in HPMECs exposed to CSE, and this difference was dose- and time-dependent. A higher concentration of PECAM EMPs inhibited the cell growth and proliferation of HPMECs and induced the apoptosis of HPMECs in a time-dependent manner. In addition, the expression of p-Akt decreased gradually as the exposure time to PECAM EMPs increased and this could be partly reversed by EUK-134. These findings suggest that PECAM EMPs may promote the apoptosis of HPMECs in COPD by de-activating the Akt pathway, and this effect can be attenuated, in part, through regulation by an antioxidant (EUK-134) (Figure 2).

EMPs play a vital role in cell-to-cell communication and can be used as a biomarker to distinguish different stimulating factors, disease status, and prognosis. In our study, EMPs phenotypes including VE-cadherin EMPs, PECAM EMPs and E-selection EMPs were more secreted in stable COPD patients compared with non-COPD volunteers, in agreement with a previous study^7^. The release of PECAM EMPs both in COPD patients and in HPMECs exposed to CSE were elevated. PECAM is an efficient signaling molecule and is now known to have diverse roles in inflammation, apoptosis, and vascular biology^25^. EMPs expressing PECAM are phenotypic for endothelial cell apoptosis^26^. In COPD patients and animal models, endothelial apoptosis plays an important role in the pathogenesis of disease^27^. In our study, the endothelial apoptosis rate increased as the concentration of PECAM EMPs increased. Moreover, apoptotic related proteins such as caspase 3/9 increased in a time-dependent manner with exposure to PECAM EMPs. Under intervention with PECAM EMPs in this study, cell growth and proliferation were also inhibited in HPMECs. Some studies propose that EMPs could be a potential biomarker and pathogenic determinant of COPD, which can indicate disease severity, while the detailed mechanisms have not been revealed^28^.

This is the first study, of which we are aware, to demonstrate that the Akt pathway is involved in endothelial apoptosis caused by PECAM EMPs. Akt is activated by phosphorylation and is known to directly and indirectly regulate the functions of apoptosis-related proteins and, consequently, protects cells from apoptosis^29^. The Akt pathway plays a critical role in the pathogenesis of COPD. Tsai et al.^30^ found some genes of the Akt signaling pathway to be associated with apoptosis and cell proliferation in COPD. In our study, although total Akt did not change, p-Akt in HPMECs was reduced gradually as the PECAM EMPs exposure time increased. These results imply that PECAM EMPs block the phosphorylation of Akt and then promote apoptosis in HPMECs, resulting in the progression of COPD.

The Akt pathway has previously been shown to be associated with oxidative stress and cell apoptosis, and can be mediated by antioxidants^31^. The antioxidant EUK-134 has been identified as protective against loss of Akt phosphorylation in unloaded skeletal muscle^16^. In our study, we determined that the apoptosis rate was decreased and the expression of p-Akt was increased in HPMECs after pre-intervention with EUK-134 compared with the PECAM EMPs group. By contrast, compared with the control group, the efficacy of EUK-134 was not obvious. These results indicate that the apoptosis of HPMECs induced by PECAM EMPs was partly ameliorated by EUK-134 through the Akt pathway, which suggests that the optimal intervention time and dose of EUK-134 are worth exploring in the future, or other antioxidants such as N-acetylcysteine or Tempol can be used to replace EUK-134 to perform additional studies. PECAM (CD31^+^), as the membrane antigen of EMPs, is known to contain 12 Ser residues and 4 Thr residues, which are phosphorylation sites^32^. Moreover, Akt activation depends on phosphorylation of Ser (473) and Thr (308) residues on it. We speculate that the PECAM EMPs modulate Akt phosphorylation through the competitive combination of Ser and Thr residues, ultimately leading to apoptosis; more experiments *in vivo* and *in vitro* are needed to identify the other pathways of apoptosis stimulation by PECAM EMPs.

### Limitations

There are some limitations in our study. Firstly, there are many components in PECAM EMPs, such as DNA, lipids, and proteins, and we have not determined which one plays the major role. This aspect will be a prime focus of future investigations. Secondly, we have observed that EUK-134 participated in the regulation of Akt phosphorylation which may inhibit the apoptotic response to PECAM EMPs. However, the detailed mechanistic pathways have not been identified. Future studies will need to investigate whether the Akt pathway combined with other molecules, such as glycogen synthase kinase-3 and mammalian target of rapamycin, contributes to COPD pathogenesis. Lastly, our studies were only performed in COPD patients and *in vitro*, and more studies *in vivo* are needed, for example, using a mouse model exposed to PECAM EMPs to observe apoptosis.

## CONCLUSIONS

Our results demonstrated that PECAM EMPs positively regulate HPMEC apoptosis in COPD by decreasing Akt phosphorylation, which can be reversed by antioxidants. These novel findings open an exciting avenue for the study of the pathophysiology of COPD: perhaps promoting Akt activation with antioxidants will protect the pulmonary vascular endothelium from apoptosis.

## Data Availability

The data supporting this research are available from the authors on reasonable request.
