# Supplementary material for: PECAM EMPs regulate apoptosis in pulmonary microvascular endothelial cells in COPD by activating the Akt signaling pathway
Source: Tob Induc Dis. 2022 May 3;20:40. doi: 10.18332/tid/146959 (PMC9059265; doi:10.18332/tid/146959)
Supplement: Supplementary file 1 [file TID-20-40-s1.pdf]

PECAM EMPs regulate the apoptosis of pulmonary microvascular endothelial cells in COPD by activating Akt signaling pathway

SUPPLEMENTARY MATERIALS

METHODS

Characterization of EMPs

The method of characterization of EMPs was conducted as previously described.[7] EMP subpopulations were determined by flow cytometry in platelet-free plasma according to the expression of membrane specific antigens: VE-cadherin EMPs: CD144+ (FITC) MPs, PECAM EMPs: CD31+ (FITC) MPs, MCAM EMPs: CD146+ (PE) MPs and E-selectin EMPs: CD62E+ (PE) MPs. We defined EMPs derived from pulmonary capillary endothelial cells as von Willebrand factor (vWF)-negative EMPs because arterioles and venules in the lungs and endothelial cells in other organs are positive while alveolar capillaries are negative for vWF.[20,21] Briefly, pulmonary EMPs were defined as VE-cadherin (CD144+/vWF-) EMPs, PECAM (CD31+/vWF-) EMPs, E-selectin (CD62E+/vWF-) EMPs and MCAM (CD146+/vWF-) EMPs.

EMP phenotype analysis was performed on size (<1.0 μm) and fluorescence. Firstly, 1.0-μm calibration microspheres (Bang Laboratories, USA) were used for identification in forward (size) and side (density) light scatter plots. Microparticle levels were corrected for each correlating isotype control antibody. A total of 5,000 EMPs with 10 μl standard count microspheres (TruCount Beads, BD Biosciences, USA) were sorted directly into RNase free microtubes containing 250 μl of RNase-free PBS. EMPs were quantified by flow cytometry using Cell Quest-Pro software (FACS Arial III, BD Biosciences, USA) by investigators blinded to subject status.

Caspase activity assays

The activation of caspase 3/8/9 was evaluated by the caspase 3/8/9 activity assay kit (Beyotime Institute of Biotechnology, China) according to the standard protocol. Briefly, HPMECs were washed with ice-cold phosphate buffered saline and treated with RIPA buffer (Beyotime Institute of Biotechnology) for 30 min at 4˚C. Supernatants were centrifuged at

20,000 × *g* at 4˚C for 10 min and collected to measure the supernatant proteins using the BCA method (Beyotime Institute of Biotechnology). To measure the activity of caspase 3/8/9, 50 μg of total proteins per well were incubated separately with Ac-DEVD-pNA, Ac-IETD-pNA, and Ac-LEHD-pNA substrates to develop a yellowish color. The absorbance of the samples was measured at 405 nm with a spectrophotometric micro-plate reader (Bio-Rad, USA).

FIGURES

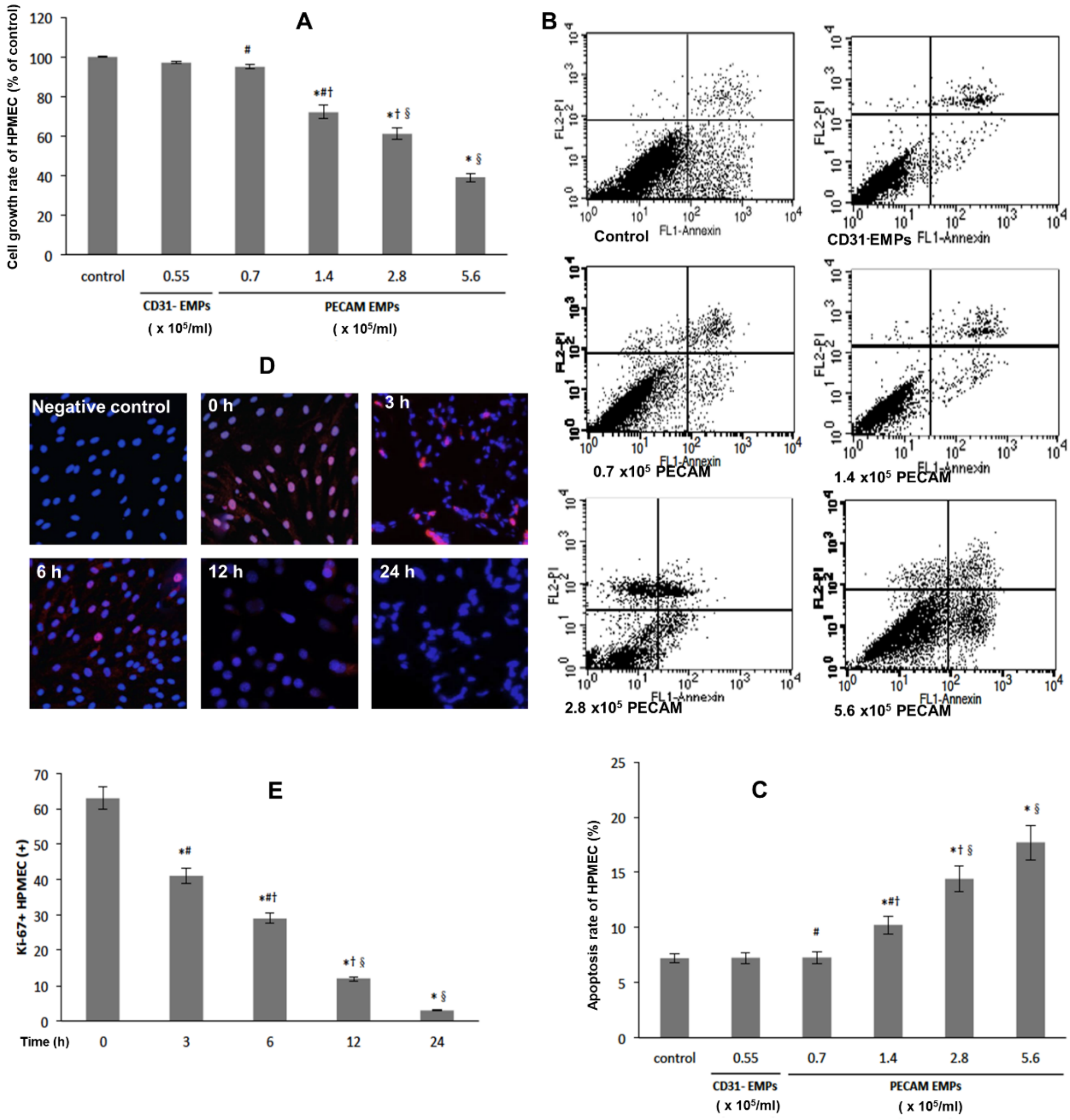

Figure S1. The cell growth, apoptosis, and proliferation of HPMECs exposed to PECAM EMPs

(A) The cell growth rate of HPMECs exposed to non-PECAM (0.55 × 10^5/ml CD31$^-$EMPs) and different concentrations of PECAM EMPs (0 ml, 0.7×10^5/ml, 1.4 × 10^5/ml, 2.8 × 10^5/ml, and 5.6

× 10^5/ml) for 24 h. The cell growth rate of HPMECs decreased gradually compared with the control group when cells were exposed to concentration higher than 1.4 × 10^5/ml PECAM EMPs (P < 0.05). **(B, C)** The apoptosis rate of HPMECs increased gradually compared with the control group when cells were exposed to concentration higher than 1.4 × 10^5/ml PECAM EMPs (P < 0.05). There was no significant difference in cell growth rate and apoptosis rate of HPMECs in CD31$^-$ EMPs and 0.7 × 10^5/ml PECAM EMPs compared with the control group (P > 0.05). **(D, E)** After treatment with 2.8 × 10^5/ml PECAM EMPs at different times (0, 3, 6, 12, and 24 hours) using Ki67 and DAPI staining, the Ki67-positive HPMECs were reduced gradually as the exposure time increased (P < 0.05). T-test was used to compare the difference between groups. *Compared with the control group, P < 0.05; #, †, § Compared with each other, P <0.05.

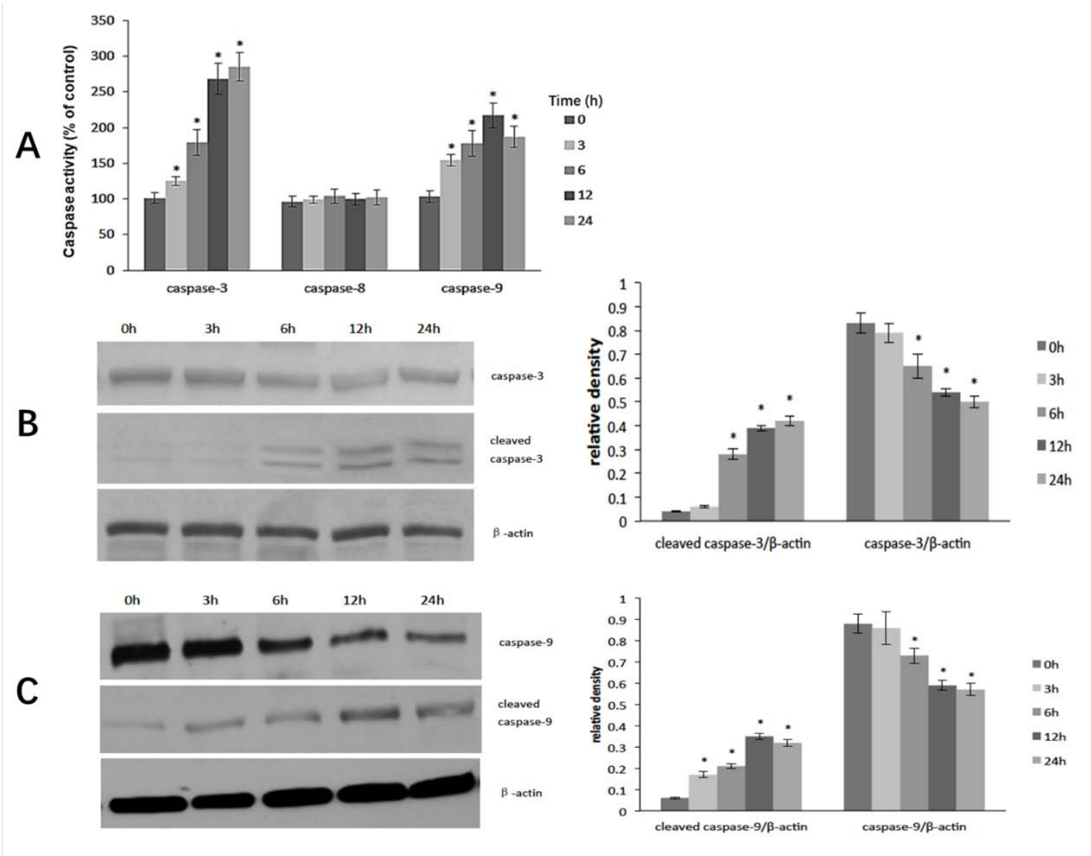

Figure S2. The activity and expression of caspase in HPMECs exposed to PECAM EMPs at different times

(A) The activity of caspase 3/9 in HPMECs exposed to PECAM EMPs increased significantly compared with the control group (0 h) as the exposure time increased (P < 0.05). There was

no significant change in caspase 8 during each period ($P > 0.05$). **(B, C)** The expression of cleaved caspase 3/9 and caspase 3/9 in HPMECs exposed to 5.6 x 10^5/ml PECAM EMPs for 0, 3, 6, 12, and 24 h. The cleaved caspase 3 increased gradually after 6 h, while caspase 3 decreased slowly. The cleaved caspase 9 increased significantly after 3 h compared with the control group ($P < 0.05$), while caspase 9 decreased slowly after 6 h ($P < 0.05$). *T*-test was used to compare with the control group, *$P < 0.05$.

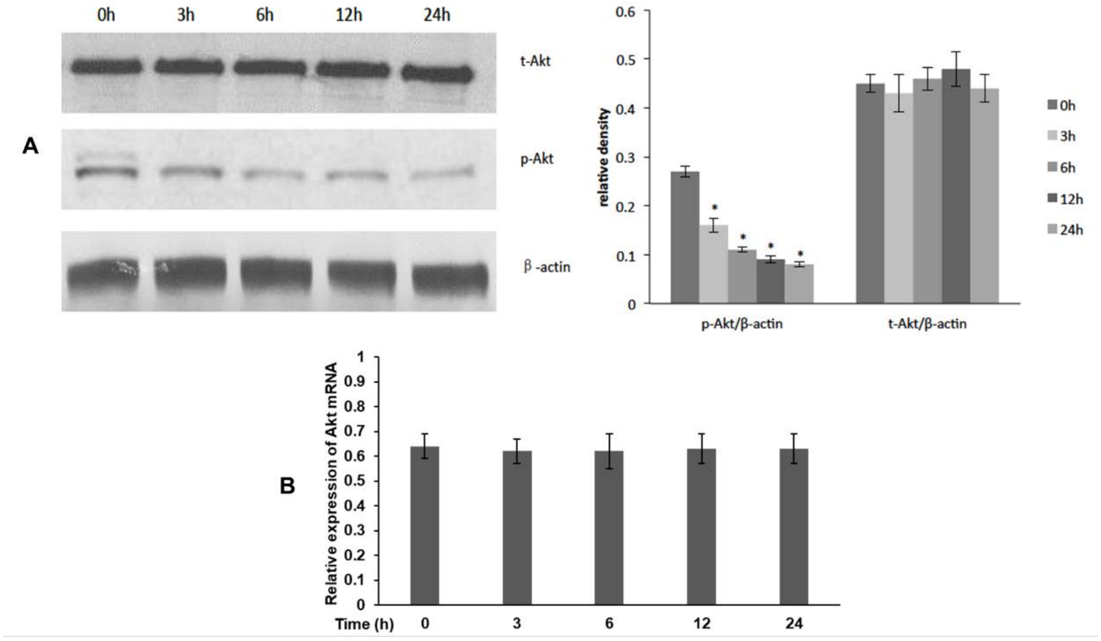

Figure S3. The expression of Akt in HPMECs exposed to PECAM EMPs at different times

(A) The expression of phospho-Akt (p-Akt) in HPMECs exposed to 5.6 x 10^5/ml PECAM EMPs at 0, 3, 6, 12, and 24 h decreased significantly compared with the control group (0 h) as the treatment time increased ($P < 0.05$). There was no significant change of total-Akt (t-Akt) ($P > 0.05$). **(B)** There was no significant change of t-Akt in HPMECs exposed to 5.6 x 10^5/ml PECAM EMPs at 0, 3, 6, 12, and 24 h using real-time PCR ($P>0.05$). *T*-test was used to compare with the control group, * $P < 0.05$.

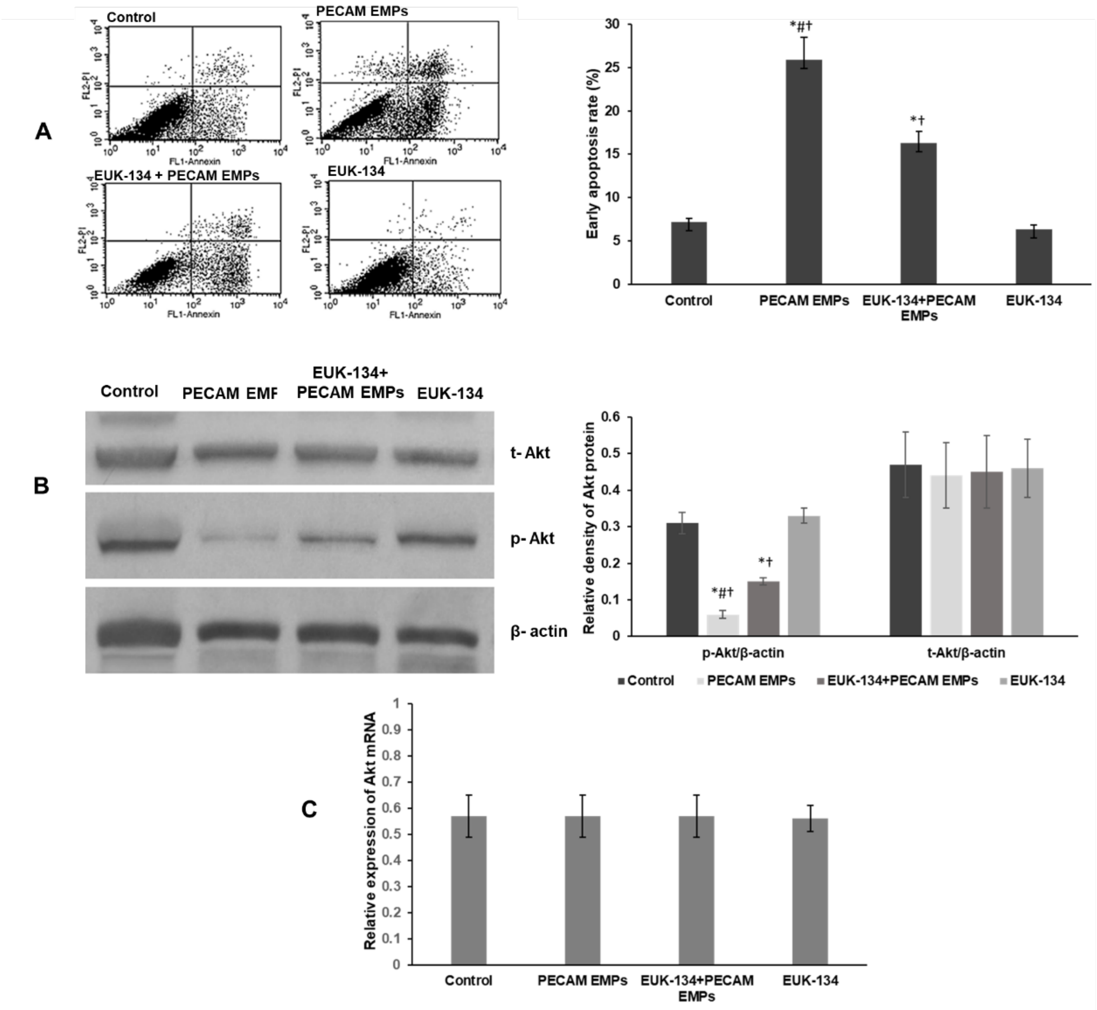

Figure S4. The apoptosis and expression of Akt in HPMECs exposed to different interventions

(A) The early apoptosis rate of HPMECs in the PECAM EMPs group and EUK-134+ PECAM EMPs group were obviously higher compared with the control group ($P < 0.05$, $P<0.05$). The apoptosis rate in the EUK-134 + PECAM EMPs group declined markedly compared with the PECAM EMPs group ($P < 0.05$). There was no difference between the control and EUK-134 groups ($P > 0.05$). **(B)** Compared with the control group, the expression of p-Akt was lower in the PECAM EMPs group and EUK-134 + PECAM EMPs group ($P < 0.05$, $P < 0.05$). When compared with the EUK-134 + PECAM EMPs group, the expression of p-Akt was lower in the PECAM EMPs group ($P < 0.05$). There was no difference between the control and EUK-134 groups ($P > 0.05$). **(C)** There were no significant differences in the mRNA expression of total Akt between groups ($P > 0.05$). *T*-test was used to compare the difference between groups. * Compared with the control group, $P < 0.05$; # Compared with the EUK-134 + PECAM EMPs

group, $P < 0.05$; † Compared with the EUK-134 group, $P < 0.05$.

© 2022 Zeng Y. et al.
